# Osteoporosis in Wilson’s disease: A large cohort study highlighting age, sex and skeletal symptoms as key risk factors for clinical surveillance

**DOI:** 10.1186/s13023-025-03910-1

**Published:** 2025-07-29

**Authors:** Ling Zhu, Bin Song, Yun Xu, Yu-long Zhu, Lei Hua, Na Nian, Long Zhang, Quan Sun, Ben-chun Xue, Yin Xu, Yong-sheng Han

**Affiliations:** 1 Institute of Neurology, Anhui University of Chinese Medicine, Hefei, 230038 China; 2 Wannan Medical College, WuHu, 241002 China; 3 Center for Xin’an Medicine and Modernization of Traditional Chinese Medicine of IHM, Anhui University of Chinese Medicine, Hefei, 230012 China; 4 Affiliated Hospital of Institute of Neurology, Anhui University of Chinese Medicine, Hefei, 230038 China

**Keywords:** Wilson’s disease, Quantitative CT, Bone mineral density, Osteoporosis, Osteopenia

## Abstract

**Background:**

Wilson’s disease (WD) is a rare disorder affecting copper metabolism that is characterized by multiple organ system damage, including the liver, brain, and eyes. Patients with WD are at risk for decreased bone mineral density (BMD). Only a few studies have investigated the relationship between WD and BMD, and there are discrepancies in the data. Therefore, we investigated the BMD status of patients with WD and analyzed the risk factors affecting the bone mass change.

**Methods:**

This retrospective cohort study selected 426 patients with WD who were admitted to a neurological hospital in Hefei, China, from January 2018 to August 2024 as study subjects. The enrolled patients were divided into the osteoporosis group (13 patients), osteopenia group (99 patients), and normal bone mass group (314 patients). The rates of prevalence of osteoporosis and osteopenia were calculated, and the risk factors of osteoporosis and osteopenia were analyzed by multivariate ordered logistic regression.

**Results:**

The prevalence of osteoporosis and osteopenia in patients with WD was 3.1% and 23.2%, respectively. Multivariate ordered logistic regression analysis demonstrated that age, male sex, and the presence of skeletal symptoms during the course of the disease were independent risk factors for osteoporosis and osteopenia in patients with WD, with odds ratio (OR) (95% confidence interval [CI]) values of 1.103 (1.074–1.134), 2.292 (1.216–4.320), and 2.675 (1.395–5.131), respectively.

**Conclusions:**

Patients with WD with older age, male sex, and skeletal symptoms during the course of the disease are prone to osteoporosis and osteopenia changes. BMD monitoring and early intervention of such patients should be strengthened clinically.

## Introduction

Wilson’s disease (WD) is a rare autosomal recessive and systemic disease primarily involving the nervous system. Epidemiological surveys have shown that the prevalence of WD in the United States and Asia is 1 in 30,000 to 1 in 50,000 [1], and the prevalence in Germany is approximately 2.03 per 100,000 [2]. A mutation in the *ATP7B* gene has been demonstrated to disrupt the excretion of copper in the liver, causing abnormal distribution and excessive accumulation of copper in the liver, brain, and other organs and tissues, thereby damaging their function and structure. Therefore, liver and brain injuries are relatively common in patients with WD, including symptoms such as elevated activity of transaminases, cirrhosis, tremors, dystonia and depression [1, 3]. Skeletal system involvement is relatively uncommon in WD. Since 2000, only seven studies have reported on WD osteoarthropathy and BMD, with two involving a large sample size. In 2005, Wang et al. reported that 23.61% of 216 patients with WD had osteoarticular symptoms, and 45.83% had decreased BMD (depending on SPA, ulna, and distal radius) [4]. In 2014, Quemeneur et al. studied 85 patients with WD and found that 51% of patients had a history of fracture and 13% of patients had changes in osteoporosis (depending on DXA, lumbar spine, and left femoral neck) [5]. Differences in the data across these studies may be attributed to variations in the study population, as well as differences in measurement sites and methods used for assessing bone density. Quantitative computed tomography (QCT) has emerged as a major technique for global osteoporosis research [6, 7]. In addition, it is more advantageous than DXA measurement [8, 9]. We used the QCT bone density measurement technology to conduct a retrospective cohort investigation on BMD in patients with WD to evaluate the current status of BMD and its influencing factors.

## Data and methods

### Study subjects

**Fig. 1:**
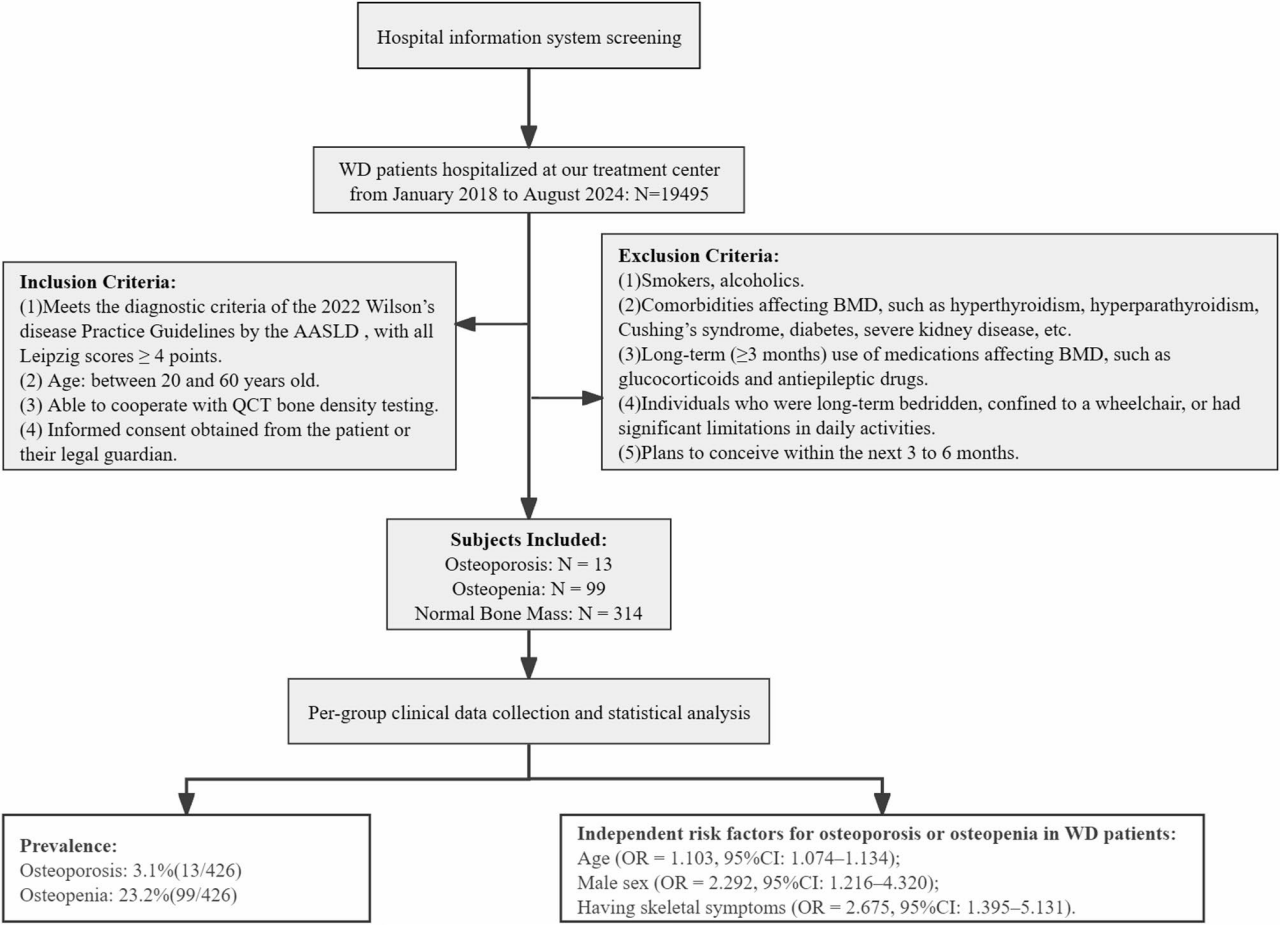
Case screening flow chart

A total of 426 WD patients hospitalized in a neurological hospital from January 2018 to August 2024 were enrolled in the study, representing 291 counties/districts across 159 cities in 25 provinces throughout China. Inclusion criteria were: ① meet the diagnostic criteria of the American Association for the Study of Liver Diseases 2022 Practice Guidelines for Wilson’s disease, with Leipzig scores ≥ 4 points [10], ② age between 20 and 60 years, ③ able to cooperate with QCT bone density testing, and ④ obtain the informed consent of the patient himself or her/his legal guardian. Exclusion criteria were: ① smokers, alcoholics, ② those having other diseases affecting BMD, such as hyperthyroidism, hyperparathyroidism, Cushing’s syndrome, diabetes, and severe kidney disease, ③ those with long-term (≥ 3 months) use of medications affecting BMD, such as glucocorticoids and antiepileptic drugs, ④ individuals who were long-term bedridden, confined to a wheelchair, or had significant limitations in daily activities, and ⑤ those who planned to conceive within the next 3 to 6 months. The flow chart of case screening is shown in Fig. 1.

## Methods

### Collection of clinical data

Information on gender, age, height, weight, liver color ultrasound classification (machine model: SiemenS-802001), corneal K–F ring, skeletal symptoms (e.g., joint deformity, pain, fracture), WD clinical classification, and treatment plan of patients was collected. Next, the body mass index (BMI) was calculated. Treatment options were divided into three groups: untreated group, monotherapy group (penicillamine treatment for at least 1 year), and combination therapy group (penicillamine treatment for at least 1 year and sodium dimercaptopropane sulfonate for at least 1 month). Assigning values to the factors are shown in Table 1.

**Table 1:** Variable values for factors affecting BMD in patients with WD

Variable	Assignment
Gender	1 = male, 2 = female
Corneal K–F ring	1 = negative, 2 = positive
Liver color ultrasound typing	1 = non-cirrhosis, 2 = cirrhosis
Manifestations of skeletal injuries	1 = yes, 2 = no
Clinical type	1 = liver type, 2 = brain type
Treatment	1 = no treatment, 2 = monotherapy, 3 = combination therapy
BMD	1 = normal bone mass, 2 = osteopenia, 3 = osteoporosis

### Biochemical index test

Patients were asked to fast for 8 h, and the venous blood was collected early the next morning. An automatic biochemical analyzer (Hitachi 7180) was used to measure the levels of hemoglobin (Hb), total bilirubin (TB), serum albumin (Alb), activity, blood urea nitrogen (BUN), creatinine (Cr), cystatin C (Cys-C), blood calcium (Ca), blood phosphorus (P), low-density lipoprotein (LDL), ceruloplasmin (Cp), and urine copper (UC) and activities of alanine aminotransferase (ALT) activity, aspartate aminotransferase (AST), and alkaline phosphatase (ALP) at 24 h before treatment. In addition, the AST/ALT ratio was calculated.

### Scale assessment

The Chinese version of the Unified Wilson’s Disease Rating Scale (UWDRS) Part 1 (Neurological Function Score) [11] was used to assess the neurological status of patients with WD. Cronbach’s alpha coefficient and KMO coefficient of the first part of UWDRS were 0.975 and 0.723, respectively, and the significance of Bartlett’s test was less than 0.001, indicating that the scale had good validity and reliability.

### BMD measurement

All patients received a Siemens double-row spiral CT machine from the United States to detect the BMD of at least two of the L1–L3 cones, and the average value was taken. The BMD results were assessed using the Chinese Quantitative CT (QCT) Osteoporosis Diagnosis Guidelines (2018), with BMD > 120 mg/cm³ classified as normal bone mass, BMD between 80 and 120 mg/cm³ as osteopenia, and BMD < 80 mg/cm³ as osteoporosis [12].

### Statistical analysis

SPSS 29.0 statistical software was used to analyze the data. Continuous variables are expressed as mean ± standard deviation (x̄±s). One-way analysis of variance was used to compare the differences between groups for data with normality, and the Kruskal–Wallis test was used to compare the differences between groups for data with non-normality. Categorical variables are expressed as counts and percentages, and differences between the groups were compared using the χ² test. Multivariate ordered logistic regression was used to analyze the risk factors of different bone mass grades. *P* < 0.05 was considered significant.

## Results

### Baseline characteristics of 426 patients with WD

Baseline characteristics of different BMD groups are depicted in Table 2. A total of 426 patients with WD aged 20 to 60 years were included, with a mean age of 31.59 ± 9.52 years. Among them, 13 patients had osteoporosis, with a mean age of 44.85 ± 9.20 years, 99 patients had osteopenia, with a mean age of 36.32 ± 10.12 years, and 314 had normal bone mass, with a mean age of 29.55 ± 8.32 years. Differences between the groups of BMD in terms of age, gender, ALP, Cr, Cys-C, P, BMD values, and skeletal symptoms were significant (*P* < 0.05).

**Table 2:** Baseline characteristics of different BMD groups

Characteristics	Total (*N* = 426)	Osteoporosis (*N* = 13)	Osteopenia (*N* = 99)	Normal bone mass(*N* = 314)	Statistical values	*P*-value
Age(yr)	29.00(24.00–38.00)	47.00(36.00–52.00)	36.00(28.00–43.00)	28.00(23.00–34.00)	H = 55.657	**<0.001**
Gender					χ² = 11.496	**0.003**
Male	248(58.22%)	8(61.54%)	72(72.73%)	168(53.50%)		
Female	178(41.78%)	5(38.46%)	27(27.27%)	146(46.50%)		
Hb (g/L)	131.00(119.00-143.00)	124.00(117.00-136.50)	133.00(121.00-143.00)	131.00(117.50–143.00)	H = 0.842	0.656
BMI	22.98 ± 4.21	24.30 ± 5.11	22.76 ± 3.84	23.00 ± 4.28	F = 0.772	0.463
TB (µmol/L)	16.80(12.98–23.03)	18.60(14.00-31.20)	17.90(13.90–22.70)	16.50(12.40–23.10)	H = 3.337	0.189
AST/ALT	1.05(0.80–1.35)	1.19(0.90–1.78)	1.08(0.84–1.34)	1.03(0.77–1.33)	H = 2.761	0.251
Alb (g/L)	41.40(37.10–44.90)	37.60(31.55-43.00)	42.40(37.40–45.50)	41.40(37.15–44.80)	H = 4.122	0.127
ALP (U/L)	89.00(67.00-115.25)	116.00(100.50-146.50)	95.00(67.00-127.00)	87.00(66.50–112.00)	H = 9.037	**0.011**
BUN (mmol/L)	5.41 ± 2.62	5.86 ± 1.20	5.79 ± 1.46	5.28 ± 2.92	F = 1.676	0.188
Cr (µmol/L)	70.85 ± 19.87	80.69 ± 25.10	75.00 ± 20.01	69.14 ± 19.35	F = 5.008	**0.007**
Cys-C (µg/dL)	89.00(75.00-105.00)	110.00(80.00-126.50)	94.00(78.00-113.00)	87.00(73.00-102.50)	H = 112.756	**0.002**
Ca (mmol/L)	2.28(2.17–2.36)	2.21(2.07–2.36)	2.30(2.23–2.37)	2.27(2.16–2.35)	H = 5.808	0.055
P (mmol/L)	1.09 ± 0.19	0.98 ± 0.11	1.05 ± 0.19	1.10 ± 0.19	F = 5.081	**0.007**
LDL (mmol/L)	2.02(1.49–2.56)	2.23(1.76–2.95)	2.06(1.51–2.69)	LDL: 1.95(1.46–2.50)	H = 3.752	0.153
Cp (mg/L)	54.70(39.60-90.98)	62.80(42.75–113.10)	52.50(38.10–83.50)	55.10(39.70-94.65)	H = 1.515	0.469
UC (µg/24 h)	246.00(146.40-388.16)	280.70(196.83–442.70)	246.30(151.25–359.50)	244.20(139.45-431.35)	H = 0.638	0.727
BMD (mg/cm3)	138.93(119.27-158.23)	72.10(62.90-77.57)	105.93(94.90-112.06)	149.27(135.77-164.92)	H = 249.460	**<0.001**
UWDRS	19.00(0.00–30.00)	22.00(8.00–30.00)	19.00(0.00–28.00)	18.00(0.00–30.00)	H = 0.423	0.809
Corneal K–F ring					χ² = 1.104	0.576
Negative	43(10.09%)	2(15.38%)	12(12.12%)	29(9.24%)		
Positive	383(89.91%)	11(84.62%)	87(87.88%)	285(90.76%)		
Liver color ultrasound typing					χ² = 1.906	0.386
Non-cirrhosis	121(28.40%)	2(15.38%)	32(32.32%)	87(27.71%)		
Cirrhosis	305(71.60%)	11(84.62%)	67(67.68%)	227(72.29%)		
Manifestations of skeletal injuries					χ² = 7.502	**0.023**
Yes	53(12.44%)	4(30.77%)	17(17.17%)	32(10.19%)		
No	373(87.56%)	9(69.23%)	82(82.83%)	282(89.81%)		
Clinical type					χ² = 0.318	0.853
Liver type	123(28.87%)	3(23.08%)	30(30.30%)	90(28.66%)		
Brain type	303(71.13%)	10(76.92%)	69(69.70%)	224(71.34%)		
Treatment					χ² = 2.163	0.706
No treatment	223(52.35%)	8(61.54%)	50(50.51%)	165(52.55%)		
Monotherapy	119(27.93%)	3(23.08%)	25(25.25%)	91(28.98%)		
Combination therapy	84(19.72%)	2(15.38%)	24(24.24%)	58(18.47%)		

### Skeletal symptoms of WD

The presence of skeletal lesions was recorded in 53 out of 426 patients with WD enrolled in the study with a ratio of 12.4% (53/426). These 52 patients had 29 osteoarticular deformities, 17 joint pains, 3 fractures, and 3 others.

### Distribution of BMD in patients with WD

**Fig. 2:**
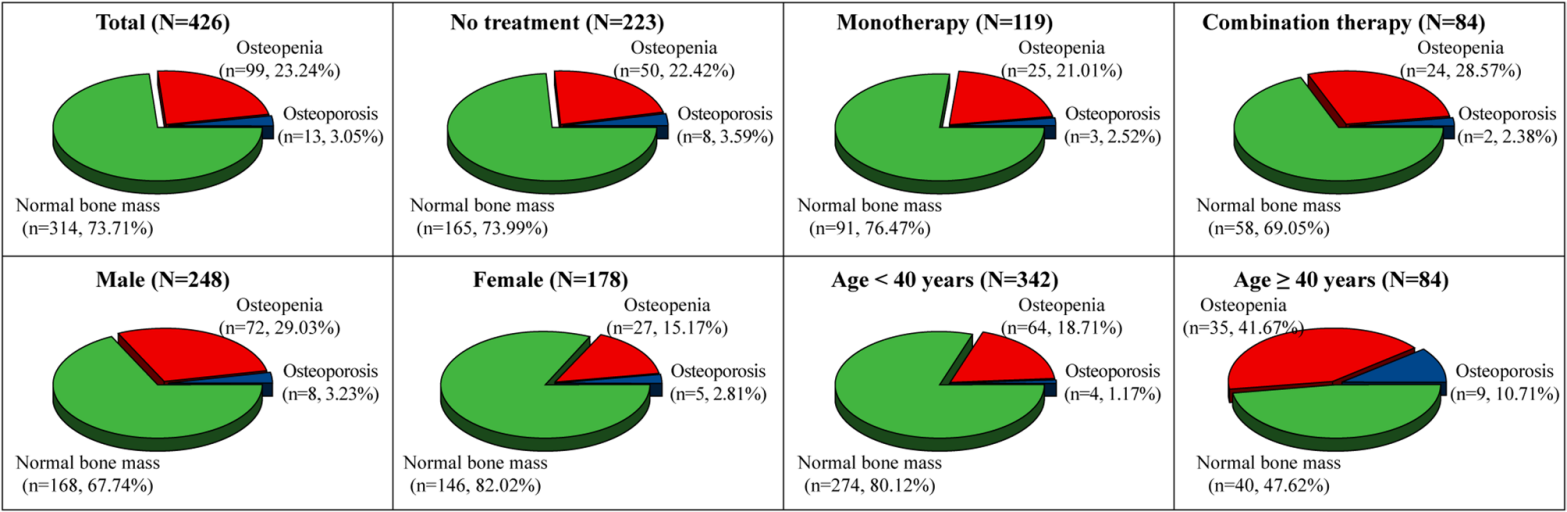
Distribution of BMD in different populations among patients with WD

Among 426 patients with WD, 13 patients were osteoporotic (3.1%, 13/426), 99 patients had osteopenia (23.2%, 99/426). The rate of bone mass abnormality (osteoporosis + osteopenia) was 26.3%, and 314 patients had a normal bone mass (73.7%, 314/426), as shown in Fig. 2. The distribution of BMD of patients with WD in other different populations is shown in detail in Fig. 2.

### Univariate logistic regression analysis of factors influencing BMD in patients with WD

The eight statistically different factors in Table 2 were used as independent variables. Univariate logistic regression analysis between each independent variable and the dependent variable was performed, which revealed that eight factors, including age (95%CI: 0.065–0.112), male sex (95%CI: 0.290–1.218), ALP (95%CI: 0.001–0.010), BUN (95%CI: 0.003–0.149), Cr (95%CI: 0.006–0.028), Cys-C (95%CI: 0.007–0.024), P (95%CI: −2.947 to − 0.599), and having skeletal symptoms (95%CI: 0.160–1.339), were associated with the occurrence of osteoporosis and osteopenia in patients with WD (*P* < 0.05), as shown in Table 3.

**Table 3:** Univariate logistic regression analysis of factors influencing BMD in patients with WD

Characteristics	β-value	SE-value	Wald χ^2^-value	*P*-value	95%CI
Age	0.088	0.012	54.188	<0.001	0.065–0.112
Gender	0.754	0.237	10.158	0.001	0.290–1.218
ALP (U/L)	0.005	0.002	4.965	0.026	0.001–0.010
BUN (mmol/L)	0.076	0.037	4.118	0.042	0.003–0.149
Cr (µmol/L)	0.017	0.005	9.534	0.002	0.006–0.028
Cys-C (µg/dL)	0.016	0.004	12.244	<0.001	0.007–0.024
P (mmol/L)	-1.773	0.599	8.758	0.003	−2.947–−0.599
Skeletal symptoms	0.749	0.301	6.206	0.013	0.160–1.339

β regression coefficient, SE standard error, Wald χ² chi-square statistic for testing β ≠ 0,

95% CI 95% confidence interval

### Multivariate logistic regression analysis of factors influencing BMD in patients with WD

**Fig. 3:**
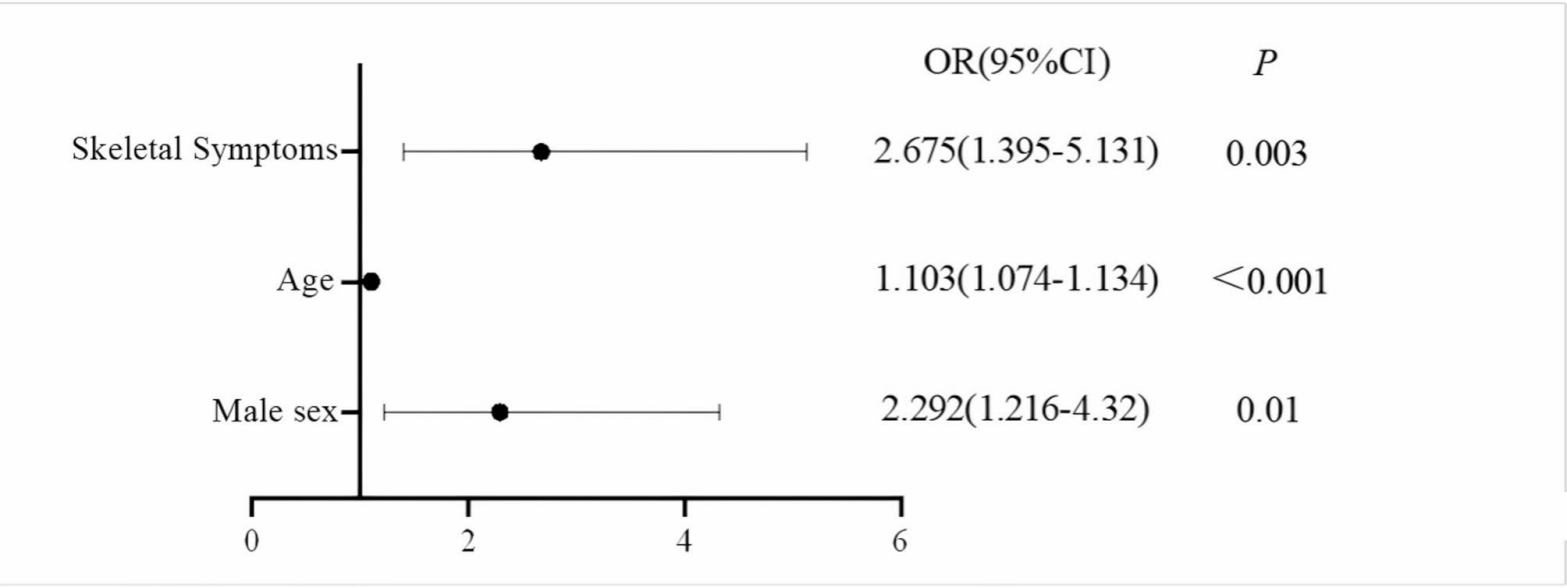
Forest plot of independent risk factors for the development of osteoporosis and osteopenia in patients with WD

Multifactorial analysis was performed with different grades of bone mineral density (1 = normal bone mass, 2 = osteopenia, 3 = osteoporosis) in patients with WD as dependent variables and factors with meaningful results from univariate analysis as independent variables. The parallelism test of χ² = 6.952 and *P* = 0.542 was greater than 0.05, which satisfied the parallel line assumption of multivariate ordered logistic regression. Multifactorial analysis revealed that age (OR = 1.103, 95%CI: 1.074–1.134), male sex (OR = 2.292, 95%CI: 1.216–4.320), and having skeletal symptoms (OR = 2.675, 95%CI: 1.395–5.131) were independent risk factors for the development of osteoporosis and osteopenia in patients with WD (*P* < 0.05), as shown in Table 4. A forest plot of independent risk factors for the development of osteoporosis and osteopenia in patients with WD is shown in detail in Fig. 3.

**Table 4:** Multivariate logistic regression analysis of factors influencing BMD in patients with WD

Characteristics	β-value	SE-value	Wald χ^2^-value	*P*-value	OR (95%CI)
Age	0.098	0.0139	50.012	**<0.001**	1.103 (1.074–1.134)
Gender	0.830	0.3233	6.585	**0.010**	2.292 (1.216–4.320)
ALP (U/L)	0.003	0.0028	1.250	0.263	1.003 (0.998–1.009)
BUN (mmol/L)	0.008	0.0516	0.023	0.880	1.008 (0.911–1.115)
Cr (µmol/L)	0.010	0.0084	1.358	0.244	1.010 (0.993–1.027)
Cys-C (µg/dL)	0.000	0.0062	0.000	0.983	1.000 (0.988–1.012)
P (mmol/L)	-0.301	0.6820	0.195	0.659	0.740 (0.194–2.816)
Skeletal symptoms	0.984	0.3323	8.769	**0.003**	2.675 (1.395–5.131)

β regression coefficient, SE standard error, Wald χ² chi-square statistic for testing β ≠ 0,

OR odds ratio, 95% CI 95% confidence interval

## Discussion

We used QCT to comprehensively evaluate BMD in patients with WD, investigate the current status of bone health, and discuss the risk factors related to osteoporosis and osteopenia. During the course of WD, bone health is a clinical problem that cannot be ignored. The associated skeletal symptoms, age, and male sex are the three warning signs of BMD reduction in patients with WD.

We investigated the true status of BMD in 426 patients with WD. The results demonstrated that the prevalence of osteoporosis was 3.1%, the prevalence of osteopenia was 23.2%, and the rate of abnormal bone mass was 26.3%. Although this result is significantly lower than that reported by Wang [4], the data presented in this study may offer improved reliability. The reasons for this are that the present study used more reasonable testing methods [8, 9] and selected the lumbar spine as the more recommended component of the test [13, 14]. The data of this study may be closer to the real-world BMD data in patients with WD. Comparison with QCT-measured BMD data from a healthy Chinese population [15] revealed that the prevalence of osteopenia among WD patients was significantly higher than that in healthy individuals in both the 20–40-year age group (RD = 14.59%, RR ≈ 4.55) and the 40–60-year age group (RD = 16.96%, RR ≈ 1.69). Among WD patients aged 20–40 years, the prevalence of osteoporosis (1.17%) was higher than in healthy controls (0%), although this difference did not reach statistical significance (RD = 1.17%, 95% CI: − 1.15–3.49%, *P* = 0.292). The point estimate suggests that WD may increase the risk of osteoporosis in younger patients, but validation in larger cohorts is required. In the 40–60-year age group, the prevalence of osteoporosis among WD patients (10.71%) was higher than in healthy controls (6.47%). This difference of 4.24% points was not statistically significant (95% CI: − 0.8–9.3%, *P* = 0.097); however, the relative risk point estimate (RR = 1.66, 95% CI: 0.83–3.30) indicates a potential trend toward increased risk, warranting regular BMD monitoring. Therefore, WD patients exhibit a significantly elevated risk of osteopenia, along with a potential trend toward increased osteoporosis risk. We recommend integrating regular QCT monitoring into the routine management of WD patients.

We speculate that BMD abnormality in patients with WD is a secondary change. The possible reasons for its occurrence are (1) Copper overload is a major pathogenic factor contributing to the development of osteoporosis in WD patients. As the primary pathological mechanism in WD, copper overload has a detrimental effect on BMD. Under normal physiological conditions, copper functions as a vital cofactor for enzymes involved in bone matrix synthesis [16]. However, excessive copper accumulation impairs skeletal development. Experimental studies have shown that increasing copper concentrations in culture media lead to a progressive reduction in the length and wet weight of chicken embryo cartilage, along with yellow discoloration of cartilage and bone, cartilage cell swelling, and diminished bone matrix formation [17]. In C57 mice, serum copper levels exhibit a negative correlation with both collagen content and bone mineral density, suggesting that copper overload may contribute to bone loss through reduced collagen synthesis [18]. Moreover, in vitro studies have demonstrated that copper inhibits the osteogenic differentiation of SD rat bone marrow mesenchymal stem cells (rBMSCs), as indicated by downregulated expression of osteogenic genes, reduced alkaline phosphatase activity, and impaired bone nodule formation, thereby disrupting skeletal remodeling during osteogenesis [19]. (2) Although WD is a genetic disorder, increasing evidence points to inflammation as a significant cofactor in its pathogenesis and bone deterioration. Wu et al. [20] reported elevated TNF-α expression in T cell subsets in WD patients. Dong et al. [21] showed upregulated mRNA expression of TNF, IL-1β, IL-6, and IL-18 in a WD mouse model, with associated inflammatory cell infiltration in multiple organs. These cytokines stimulate osteoclastogenesis by upregulating macrophage colony-stimulating factor (M-CSF) and receptor activator of nuclear factor kappa-B ligand (RANKL), leading to accelerated bone resorption [22]. Furthermore, Liu et al. [23] documented increased reactive oxygen species (ROS) in copper-overloaded mice, which intensify oxidative stress, enhance osteoclast activity, suppress osteoblast differentiation, and ultimately contribute to reduced BMD [22].

223 patients with WD in the included subjects did not receive any treatment, their prevalence of osteoporosis and osteopenia were 3.6% and 22.4%, respectively, which were not statistically different from the prevalence of osteoporosis and osteopenia of 119 patients who received monotherapy and 84 individuals who received the combination therapy (*P* = 0.706, Table 2). The differences in BMD among treatment groups did not reach statistical significance. This may be attributed to the fact that the primary mechanisms of action of the therapeutic agents (penicillamine and sodium dimercaptopropanesulfonate) do not directly affect key pathways involved in bone metabolism. Existing literature also indicates that these agents have no significant impact on calcium homeostasis or bone metabolic processes [24–26].

The occurrence of osteoporosis and osteopenia is a non-negligible skeletal pathology problem in the course of WD. We found that 12.4% of 426 patients with WD had skeletal symptoms, including joint deformity, joint pain, and fracture. This percentage was lower than the results reported by Wang [4] and Quemeneur [5]. This discrepancy may be due to our strict exclusion of known osteoporosis risk factors such as smoking [27], reduced physical activity [28], and glucocorticoid use [29]. These exclusions likely reduced the prevalence of skeletal symptoms in our cohort. Moreover, our logistic regression analysis showed that the presence of skeletal symptoms during WD increases the risk of osteoporosis and osteopenia. Osteoporosis is a skeletal disorder characterized by decreased bone mass, damage to microstructures such as trabeculae, increased bone fragility, and susceptibility to fracture [14, 30]. Early clinical symptoms are acute or chronic back pain, with further loss of bone mass, short stature, and bone deformities (such as scoliosis, hunchback, and joint deformity). Pathological fracture is the most characteristic and serious manifestation of osteoporosis [31]. While our data suggest a predictive relationship between skeletal symptoms and low BMD, reverse causality cannot be excluded. It is plausible that low BMD may predispose patients to skeletal symptoms. In this study, symptoms were reported before BMD measurements, supporting the hypothesized directionality. Nevertheless, we recognize that this temporal ordering does not establish causation. Future longitudinal studies with repeated measurements and larger samples are essential to clarify the causal nature of this association.

The present study demonstrated that age is an independent risk factor for osteoporosis and osteopenia in patients with WD. This is consistent with the findings of several studies [32–34]. Under normal physiological conditions, human bone mass increases with age, reaches a peak in late puberty, and gradually decreases thereafter [35, 36]. This change is related to the decrease in the levels of estrogen and androgen in the human body. Both estrogen and androgen inhibit bone resorption, promote bone formation, and maintain the body’s bone mass [37, 38]. With aging, the body’s estrogen and androgen levels decline, the role of bone maintenance is weakened, and the bone mass is reduced. Simultaneously, osteotoxicity due to copper overaccumulation increases with age. Thus, low estrogen and androgen levels and prolonged copper overload in older patients with WD constitute the primary reasons for the occurrence of osteoporosis and osteopenia. The risk of osteopenia and even osteoporosis should not be ignored in older patients with WD, and timely screening and reasonable monitoring are crucial. Optimizing the copper chelation regimen and maintaining the body’s copper balance are important measures to prevent osteoporosis and osteopenia.

A study of BMD in Chinese individuals reported that BMD values and the prevalence of osteoporosis were comparable and not statistically different in normal men and women between 35 and 59 years of age [39]. However, in the present study, gender was found to be different between the different bone mass groups (*P* = 0.001), and the risk of osteoporosis and osteopenia was higher in male patients than in female patients. This is consistent with the findings of Massimo [40]. Differences in BMD between genders are primarily related to sex hormone levels [37, 38]. Although literature on hypothalamic-pituitary-gonadal (HPG) axis function and sex hormone levels in WD patients is limited, hepatic injury remains a consistent feature throughout the WD disease course. Multiple studies on sex hormones in liver disease demonstrate elevated serum estrogen levels and reduced androgen levels [41, 42]. Androgens promote bone formation by acting on osteoblasts through androgen receptors (AR), thereby enhancing osteoblast proliferation and differentiation and increasing BMD [43, 44]. Additionally, a meta-analysis showed that androgen deprivation therapy (ADT) significantly reduces BMD in prostate cancer patients [45]. Consequently, hepatic injury in WD may result in reduced androgen levels, impairing androgen-mediated osteogenesis and compromising BMD. This mechanism may contribute to the elevated risk of osteoporosis and osteopenia observed in male WD patients.

### Limitations

This study has the following strengths: our study features a substantial sample size—among the largest in current literature—and utilized the more advantageous QCT to investigate BMD changes.

Some limitations should be acknowledged for this study: (1) lack of mechanistic insights: The lack of sex hormone profiles and bone turnover biomarkers limits mechanistic exploration of the linkage between skeletal symptoms and BMD. Future multicenter studies should incorporate these measurements to clarify copper–bone endocrine interactions. (2) limited generalizability: Single-center design risks regional selection bias despite geographic diversity, requiring multicenter validation with standardized environmental data. (3) subgroup analysis constraints: The small subgroup of patients with osteoporosis (*n* = 13) may result in unstable effect estimates in the regression analysis and limit the generalizability of our conclusions. Accordingly, findings in this subgroup should be interpreted with caution. (4) restricted applicability: Findings primarily reflect non-smoking young/middle-aged WD patients; extension to elderly, pediatric, or smoking populations needs prospective confirmation.

## Conclusions

Our study indicates that young and middle-aged WD patients (20–60 years) with WD exhibit a significantly increased risk of osteopenia, coupled with a rising trend toward osteoporosis. Notably, older age, male sex, and the presence of skeletal symptoms heighten these risks. These findings support implementing structured bone health surveillance in WD management protocols, specifically targeting high-risk subgroups (males ≥ 40 years with skeletal symptoms) through annual QCT assessments to mitigate these risks. We hope that our findings may contribute useful reference information for future investigations and research in this area.

## Data Availability

The datasets used and/or analysed during the current study are available from the corresponding author on reasonable request.
